# Clinical differences between outpatients with and without internet addiction and emotional disorders: a prospective naturalistic outcome study

**DOI:** 10.3389/fpsyt.2024.1357477

**Published:** 2024-03-22

**Authors:** Jiaqi Zhou, David H. Rosmarin, Steven Pirutinsky

**Affiliations:** ^1^ Shanghai Key Laboratory of Mental Health and Psychological Crisis, Affiliated Mental Health Center (ECNU), School of Psychology and Cognitive Science, East China Normal University, Shanghai, China; ^2^ Center for Anxiety, New York, NY, United States; ^3^ McLean Hospital/Harvard Medical School, Belmont, MA, United States; ^4^ Graduate School of Social Work, Touro College, New York, NY, United States

**Keywords:** internet addiction, emotional disorder, depression, anxiety, comorbidity, adults

## Abstract

**Background/Objective:**

as internet use becomes increasingly ingrained in contemporary society, internet addiction (IA) has emerged as a global public health concern. There is ongoing debate regarding whether IA represents a distinct psychological disorder or a secondary manifestation of other existing disorders. This study aimed to examine the pathological relationship between IA and emotional disorders (ED).

**Method:**

this study compared pre-treatment characteristics and treatment process of three groups of patients (N=1292) in a naturalistic treatment setting: IA only, ED only, and comorbidity of IA and ED.

**Results:**

the IA only group differed from the other groups by reporting the highest levels of life satisfaction, adaptive emotion regulation, as well as risk behavior urges at intake. In addition, the IA only group displayed the lowest level of depressive and anxiety symptoms throughout the treatment.

**Conclusion:**

our findings contribute to a better understanding of the discreteness of IA as a potential psychological disorder and inform more effective treatment strategies for IA and its comorbid conditions.

## Introduction

As internet use becomes increasingly ingrained in contemporary society, excessive and problematic use of the Internet, termed as internet addiction (IA), has become a global public health issue (1, 2). As IA’s global significance grows, there is ongoing debate on whether IA is a discrete psychological disorder or a secondary manifestation of another existing disorder (3–5). On one hand, the high rate of comorbidity between IA and other psychological disorders, especially emotional disorders, points toward IA being a symptom manifestation of an underlying disorder (ED; 4, 6–10). On the other hand, many scholars have proposed different versions of diagnostic criteria for IA (11–14), supporting IA as a discrete psychological disorder. Examining treatment response of clinical cases of IA, ED, and their comorbidity separately in a naturalistic setting may provide insights not only to clarify IA’s pathological discreteness, but also to design more effective treatments for IA and related comorbid disorders.

A comparison of cases of IA, ED, and comorbidity of the two in pre-treatment condition can contribute to a more comprehensive pathological classification of IA. For example, while literature has consistently shown an association between ED and lack of life satisfaction (15, 16), the corresponding link between IA and life satisfaction is unclear. Even though research has also indicated a negative relationship between IA and life satisfaction (17, 18), one study suggested that the link is absent among male compared to female participants based on a sample of commuters (19). Similarly, compared to a sample in the US, an equivalent sample of Italian participants did not demonstrate an association between IA and life satisfaction (20). In other words, this association between IA and life satisfaction may not be as universal as the one between ED and life satisfaction, suggesting the possibility of pathological discreteness between IA and ED.

Another pathological characteristic that might differentiate IA and ED is emotion regulation strategies. Prior research has shown that ED is highly associated with maladaptive emotion regulation strategies, especially suppression and lack of cognitive reappraisal (for reviews, see 21, 22). Similarly, emotion dysregulation also plays a role in maintaining IA symptoms as maladaptive emotion regulation strategies, since engaging in online activities often serves as a distraction in response to stressors and painful emotions (for a review see, 23). In other words, IA and ED may differ in terms of the primary specific maladaptive emotion regulation strategies.

In addition to their differences with regard to emotion regulation, examining whether risk behaviors can differentiate IA and ED may further clarify the potential conceptualization of IA as a discrete psychological disorder. A growing number of research has indicated the close association between IA and impulsivity traits (24–26), which is not the primary diagnostic symptom for emotional disorder among adults (DSM-5; 27). Therefore, compared to patients with ED, patients with IA may be more likely to display impulsivity traits, such as urges to harm self and others or use drugs and alcohol. Besides risk behaviors in IA and ED patients, analyzing the pre-treatment characteristics in individuals with comorbid IA and ED can provide deeper insights into the complex pathological interplay between these two conditions.

Furthermore, examining differences in treatment process between IA, ED, and their comorbidity may provide additional confirmation of IA as a distinct pathology. A few studies have demonstrated that psychotherapy and medicine for ED can be effective to treat patients with IA and ED comorbidity (for a review, see 28). However, the treatment may not be equally effective for IA and ED across patients. One study examining the efficacy of psychotherapy for depressed adolescents with IA showed that cognitive behavioral therapy (CBT) reduced symptoms of IA but not depression (29). Another study showed that while combined treatment of CBT and medicine reduced both levels of IA and depression for their sample of male adult patients, only the reduction in IA was maintained at the 4-week follow up, while depressive symptoms recurred (30). The inequality of treatment effectiveness for IA and ED may be a result of IA symptoms being an obstacle for ED depression. A study in a naturalistic setting indicated that depressed patients with a higher level of IA not only showed a more slowed down process of symptom reduction but terminate treatment with a higher level of depression, compared to patients with less IA symptoms (31). These findings suggest that comorbid cases may be less responsive to treatment compared to IA and ED cases separately, a result that would be unexpected if IA and ED were pathologically equivalent.

While prior research of IA in treatment settings has primarily examined comorbid cases only or relied on healthy controls as a comparison (for a review, see 28), the present study explored the potential difference in treatment progress for adult patients with IA only, ED only, and comorbidity of the two in a naturalistic outpatient clinic setting. Specifically focusing on IA and ED individually along with comorbid cases can reveal nuances in pre-treatment factors, as well as their response to typical psychological therapy among three groups. We first explored differences across the three groups on symptom level, life satisfaction, cognitive reappraisal, and risk factors. We hypothesized that among the three groups, patients with IA only would show the highest level of functionality, whereas patients with comorbidity would show the lowest level of functionality before onset of treatment. We further hypothesized that compared to patients with IA only and ED only, patients with comorbidity might be less responsive to treatment as indicated by slower and/or less change in ED symptoms over the course of treatment.

## Method

### Procedures & participants

Data was collected between September 2018 to April 2022 from all consenting adult patients presenting to the offices of a private New York and Boston based outpatient clinic. At intake and at each treatment session, patients completed self-report measures of depression (described below) using Psych-Surveys™ software. At intake, all patients also received a general psychosocial interview as well as the Miniature International Neuropsychiatric Interview (32) to assess for DSM-V diagnoses, which were conferred in consultation with licensed clinical psychologists during weekly clinical rounds meetings. In accordance with clinic procedures, psychotherapy was of a Cognitive-Behavioral and/or Dialectical-Behavior Therapy (CBT/DBT) nature. No uniform treatment protocol was enforced across all patients, acknowledging the variation in patient needs in real-world clinical environments, but core principles of CBT and DBT were consistently applied. Treatment was provided by licensed psychologists, licensed social workers, and doctoral interns and postdoctoral fellows who received weekly individual as well as group supervision and additional consultation under licensed psychologists as needed throughout treatment. During the these supervision and consultation meetings, clinicians engaged in peer discussions of case management and treatment planning while adhering to evidence-based practice protocols, ensuring the consistency and quality of therapy delivered across the diverse caseloads. These meetings were structured to facilitate collaborative analysis of therapeutic progress, address any potential ethical concerns, and provide a platform for the exchange of clinical expertise and support among the treatment team.

Inclusion criteria in the current analyses were as follows: 18 years of age or older; “moderate” levels of depression or anxiety, or self-reported dependent users of the Internet (described below). Our initial sample included 1292 patients ranging in age from 18 to 84 years (**Table 1**), whose intake data were used for part of the initial analyses. We categorized patients into three groups based on their PHQ-9, GAD-7, and IA scores (see in the “Measures” section below): patients who reported to be dependent on the Internet but did not meet threshold for “moderate” level of depression or anxiety (IA only, n=94); patients who reported not to be at risk for dependence on the Internet and did meet threshold for “moderate” level of depression or anxiety (ED only; n=705); patients who reported to be at risk for dependence on the Internet and meet threshold for “moderate” level of depression or anxiety (Comorbid, n=493).

**Table 1:** Demographic & clinical characteristics of the sample (*n* = 1226).

Sample Characteristics	%	*M*	*SD*
Age		31.35	12.04
Total recorded sessions		7.36	13.54
Depression Intake (PHQ-9)		13.89	6.40
Anxiety Intake (GAD-7)		14.62	4.70
Internet Addiction		2.65	2.33
Gender			
female	62.5%		
male	34.4%		
nonbinary	2.4%		
undefined	.6%		
Income			
<$25,000	26.9%		
$25,000 - $50,000	11.5%		
$50,001 - $75,000	10.8%		
$75,001 - $100,000	12.3%		
$100,001 - $130,000	9.9%		
$130,000 - $250,000	14.9%		
>$250,000	13.4%		
unreported	.2%		
Ethnicity			
Black/African-American	3.1%		
Asian-American	3.7%		
White	80%		
Latino or Hispanic	3.8%		
Multi-racial	2.9%		
Other	3.9%		
Rather Not Say or unreported	2.6%		

For the remaining analyses that examined group differences in treatment process, we excluded patients who had less than three recorded total sessions. Patients who were included into the trajectory analyses did not differ from those excluded on age, IA score, or GAD-7 score, but they did differ on PHQ-9 score (t_(1,1289.7)_=6.48, *p*=.01; *M*_included_=13.43, SD_included_=6.27; *M*_excluded_=14.34, SD_excluded_=6.57), suggesting that patients with a higher level of depression were either less likely to comply with session survey completion or more likely to terminate treatment immaturely. This exclusion resulted in a 53.3% attrition rate, leaving 604 patients (IA only=46; ED only=342; comorbid =248) who over the course of the study attended a total of 8640 sessions.

### Measures

For demographic information, we assessed patients’ age, gender, income, and ethnicity using a standardized questionnaire administered at intake. For income, we measured patients’ annual income on an interval scale ranging from <$25,000 to >$250,000.

Depression was measured using the Patient Health Questionnaire Depression Scale (PHQ-9; 33) a nine-item self-report measure of depressive symptoms. The PHQ-9 yields a single total score between 0 and 27, and can be interpreted using four validated levels of depression severity: “Minimal” (0–4); “Mild” (5–9); “Moderate” (10–14); “Moderately Severe” (15–19) and “Severe” (20–27; 33).

Anxiety was measured using the General Anxiety Disorder-7 (GAD-7; 34), a seven-item self-reported measure of anxiety symptoms. The GAD-7 yields a single total score between 0 and 21, and can be interpreted using four validated levels of anxiety severity: “Minimal” (0–4); “Mild” (5–9); “Moderate” (10–14); and “Severe” (15–21; 34).

IA was assessed with Young’s Diagnostic Questionnaire (YDQ; 35), including eight self-report items detailing criteria of problematic internet use with binary response options (Yes or No). The measure resulted in a total score from 0-8. Three or more ‘yes’ responses to the eight questions indicate being at risk for dependence (35).

Satisfaction with life was measured by the Satisfaction with Life Scale (SWLS; 36), a 7-point Likert style response scale. The possible range of scores is 5-35, with a score of 20 representing a neutral point on the scale. Scores between 5-9 indicate the respondent is extremely dissatisfied with life, whereas scores between 31-35 indicate the respondent is extremely satisfied. The scale has demonstrated high internal consistency (alpha=.89) and test–retest reliability (r=.84, 37).

Emotion regulation was measured by the Emotion Regulation Questionnaire (ERQ; 38), a 10-item self-report measure of an individual’s habitual use of expressive suppression and reappraisal to regulate emotion. This measure is composed of a 4-item expressive suppression subscale and a 6-item cognitive reappraisal subscale. Each item is rated on a 7-point Likert scale (1 = strongly disagree; 7 = strongly agree). Subscales were summed, with higher scores indicating greater use of the strategy. The ERQ has demonstrated high internal consistency (.79 for reappraisal and.73 for suppression) and test–retest reliability of.69 for both subscales (38).

Other psychological risk factors were measured by the following questions on a 7-likert scale (1=not at all; 7=very strong): (1) How has your *stress level* been? (2) How has your *anger level* been? (3) How has been your *urge to harm yourself*? (4) How strong has been your *intent to kill yourself*? (5) How strong has been your *urge to use drug or alcohol*? (6) How strong has been your *intent to hurt another person*?

The intake survey included all the measurements mentioned above, whereas the session survey only included PHQ-9, GAD-7, levels of stress, suicide intent, and urge to use drug or alcohol.

### Data analytic plan

For initial analyses, we examined the associations between group (IA only vs. ED only vs. Comorbid) and demographic variables, including age, gender, income and ethnicity, using various factorial analyses. We then examined the associations between group and life satisfaction, emotion regulation, and psychological risk factors at intake. We further examined the trajectory of change in anxiety and depressive symptoms over the course of treatment using multilevel growth curve models (39). Models were estimated using restricted maximum likelihood estimation with the lme4 library (40) and coefficients tested with the lmerTest library (41) in the R programming language (42).

Initial examination of treatment trajectories suggested that change in anxiety and depression scores was best described by a cubic pattern with an initial period of rapid decline followed by a longer period of slower improvement. Specifically, cubic models fit significantly better than linear models (ΔAIC_anxiety_ = 648, χ2(2) = 651.93, *p* <.001; ΔAIC_depression_ = 409, χ2(1) = 413.08, *p* <.001), and sequentially adding random cubic effects (ΔAIC_anxiety_ = 407, χ2(4) = 414.82, *p* <.001; ΔAIC_depression_ = 359, χ2(1) = 366.88, *p* <.001) significantly improved model fit. This model represented our baseline model of treatment trajectory for both anxiety and depression symptoms. Building on this baseline model, we further examined the effect of group and its interaction with slopes on clinical symptoms.

### Ethics

This study was completed based on unidentifiable data and was approved by the Institutional Review Board of Touro College (IRB Exempt Protocol Number: IRB1-2023-003).

## Results

### Analyses of variables at intake

Demographic and clinical characteristics of the entire sample are presented in **Table 1**, and the means and standard deviations of variables across groups are presented in **Table 2**. Based on the full sample (n=1226), patients’ age at intake (*F*_(2, 285.58)_ = 41.51, *p* <.001), income level (*F*_(2, 264.42)_ = 9.49, *p* <.001) significantly differed across groups. Post-hoc Tukey tests showed that patients in the IA only and Comorbid group were significantly younger, and reported a lower level of income than those in the ED only group (*ps*<.01). Ethnicity, gender, and total recorded number of session did not differ across groups.

**Table 2:** Means and Standard Deviations (in parentheses) of each intake variable across groups.

	Full Sample				Sample for Treatment Trajectory Analyses			
	IA only (n=94)	ED only (n=705)	Comorbid (n=493; C)	Post-hoc Tests Results†	IA only (n=46)	ED only (n=342)	Comorbid (n=248; C)	Post-hoc Tests Results†
Age	28.53*(8.31)	33.97*(13.55)	28.15*(10.51)	IA-ED; ED-C	28.90* (8.90)	34.16* (13.92)	27.99* (9.46)	IA-ED; ED-C
Income	3.05*(2.00)	3.87*(2.18)	3.46*(2.20)	IA-ED; ED-C	3.16*(2.10)	3.80*(2.19)	3.41*(2.15)	
Total recorded number of sessions	7.47(14.79)	7.98(15.14)	6.45(8.84)		12.70(17.68)	15.07(19.34)	11.73(13.51)	
Anxiety at intake	5.66*(2.04)	15.18*(4.04)	15.52*(4.22)	IA-ED; IA-C	5.82*(2.19)	14.94*(3.97)	15.69*(4.07)	IA-ED; IA-C
Depression at intake	5.01*(2.60)	14.41*(6.05)	14.84*(6.22)	IA-ED; IA-C	4.98*(2.60)	13.76*(5.91)	14.49*(6.16)	IA-ED; IA-C
Life Satisfaction	21.83*(7.55)	16.23*(7.71)	15.61*(7.42)	IA-ED; IA-C	22.43*(7.83)	17.18*(7.83)	16.10*(7.76)	IA-ED; IA-C
Cognitive Reappraisal	26.19*(6.82)	22.39*(7.25)	20.97*(7.35)	IA-ED; IA-C;ED-C	25.93*(6.76)	23.00*(7.23)	20.83*(7.31)	IA-ED; IA-C;ED-C
Expression Suppression	12.54(5.14)	13.75(5.62)	13.64(5.45)		12.79*(5.01)	14.01*(5.68)	13.68*(5.40)	
Stress	5.54(1.34)	5.41(1.37)	5.48(1.26)		4.62*(1.28)	5.62*(1.17)	5.83*(1.23)	IA-ED; IA-C
Anger	3.59*(1.71)	3.07*(1.65)	3.51*(1.70)	ED-IA;ED-C	2.64*(1.49)	3.38*(1.72)	3.33*(1.69)	IA-ED; IA-C
Urge to harm self	2.32*(1.63)	1.83*(1.50)	2.18*(1.70)	ED-IA;ED-C	2.16*(1.69)	1.86*(1.54)	2.25*(1.80)	ED-C
Suicide Intent	1.93*(1.42)	1.45*(1.05)	1.69*(.34)	ED-IA;ED-C	1.18*(.76)	1.60*(1.27)	1.69*(1.39)	IA-ED; IA-C
Urge to hurt others	1.21*(.70)	1.11*(.51)	1.32*(.94)	ED-C	1.13*(.50)	1.12*(.54)	1.36*(.95)	ED-C
Urge to use drugs or alcohol	3.10*(1.91)	2.24*(1.77)	2.58*(1.84)	IA-ED; IA-C;ED-C	1.21*(.71)	2.00*(1.61)	2.13*(1.70)	IA-ED; IA-C

*indicates that there was significant difference across the three groups based on ANOVA tests (p<.05).

† results indicate that posthoc tests showed significant difference between the two groups. e.g., “IA-ED” means that there was significant difference between the IA and ED groups.

For clinical symptoms, patients’ anxiety and depression levels significantly differed across groups (*F*_(2, 350.76)_ = 797.52, *p* <.001; *F*_(2, 394.19)_ = 443.64, *p* <.001 respectively). Specifically, post-hoc Tukey tests indicated that patient in the IA only group reported a significant lower level of both anxiety and depression symptoms compared to those in the other groups (*p*s<.01), but the ED and Comorbid groups did not differ from each other.

Patients’ life satisfaction level also significantly differed across groups (*F*_(2, 259.29)_ = 27.14, *p* <.001), and post-hoc Tukey tests showed that the IA group reported a significant higher level of life satisfaction than the other groups (*p*s<.01). Again, the ED and Comorbid groups did not differ from each other.

For patients’ emotion regulation skills, cognitive reappraisal significantly differed across groups (*F*_(2, 262.77)_ = 23.21, *p* <.001) while expressive suppression did not (*p*>.05). Post-hoc Tukey tests indicated that all three groups significantly differed from each other on cognitive reappraisal (*p*<.01), with patients in IA only group showing the highest level, followed by the ED only group and then the Comorbid group.

In terms of risk items, while three groups did not differ on stress level (*p*>.05), they did significantly differ on anger level (*F*_(2, 256.79)_ = 11.27, *p* <.001), urge to harm self (*F*_(2, 254.57)_ = 9.04, *p* <.001), suicide intent level (*F*_(2, 244.27)_ = 9.12, *p* <.001), and intent to hurt others (*F*_(2, 240.28)_ = 11.08, *p* <.001), and urge to use drug (*F*_(2, 254.43)_ = 11.4, *p* <.001). Post-hoc Tukey tests showed that the ED only group reported a significantly lower level of anger, urge to harm self, and suicide intent than the other groups (*p*s<.05). In addition, ED only group showed a significantly lower level of urge to harm others compared to the Comorbid group (*p*<.05). Finally, all three groups significantly differed from each other on urge to use drug (*p*s<.05), with the IA only group showing the highest level of urge to use drug, followed by the Comorbid group and then the ED only group.

When repeating the same analyses on the subset sample for treatment trajectory analyses (n=604), most results aligned with those previously reported (See **Table 2**), except that: (1) the difference in income across groups was no longer significant in post-hoc tests; (2) compared to both ED only and Comorbid groups, the IA only group showed significant lower levels of stress, anger, suicide intent, and urge to use drugs or alcohol; (3) the difference in urge to harm self between IA only and ED only groups was no longer significant.

### Analyses of treatment trajectory

To explore whether the treatment trajectory differed across groups, we estimated a series of conditional multilevel regression models building on the unconditional analysis described above. Model comparisons are reported in **Table 3**. For both treatment trajectories of anxiety and depression symptoms, results indicated that the addition of group as a main effect (M4) as well as its interactions with liner, quadratic, and cubic slopes (M5) significantly improve model fit. Thus we accepted M5 as the best fitting model for anxiety and depressive symptom trajectories.

**Table 3:** Conditional multilevel regression models for IA and treatment length and their interaction.

Models	Anxiety Symptom					Depression Symptom				
	*df*	*AIC*	*-2LL*	*X^2^*	*p*	*df*	*AIC*	*-2LL*	*X^2^*	*p*
M1: Random Intercept Only	4	48075	-24033			4	49897	-24945		
M2: Fixed Slopes	6	47427	-23708	651.93	<.001	6	49488	-24738	413.08	<.001
M3: Random Slopes	15	45644	-22807	1801.1	<.001	15	47677	-23824	1829.2	<.001
M4: Group	17	46206	-23086	153.26	<.001	17	47593	-23780	87.53	<.001
M7: Group × Slopes	23	46177	-23066	40.64	<.001	23	47577	-23766	28.00	<.001

Model coefficients and graphs of the most fitted models are shown in **Table 4**. For the main and interaction effects of group, the ED only was used as an anchor subgroup to be compared with the other two subgroups (IA only and Comorbid). In other words, a significant coefficient corresponding to a subgroup indicates a significant difference in the outcome variable compared to ED only. For both the anxiety and depressive symptom trajectories, the IA only and Comorbid subgroups significantly differed from the ED only subgroup overall, and the group interacted with slopes.

**Table 4:** Coefficients for the most fitted models-only including the significant effects.

Fixed Effects	Anxiety Symptom				Depression Symptom			
	*Unstandardized* *Coefficient*	*SE*	*t*	*p*	*Unstandardized* *Coefficient*	*SE*	*t*	*p*
Intercept	13.59	0.22	61.73	<.001	12.85	.31	42.0	<.001
Linear Slope	-129.95	7.96	-16.33	<.001	-103.14	8.12	-12.70	<.001
Quadratic Slope	756.55	63.73	11.87	<.001	571.03	62.01	9.21	<.001
Cubic Slope	-1248.45	124.08	-10.06	<.001	-924.78	120.08	-7.70	<.001
IA-only	-7.54	0.57	-13.24	<.001	-7.78	0.79	-9.86	<.001
IA-only × Linear Slope	99.69	21.45	4.65	<.001	70.72	21.92	3.23	<.01
IA-only × Quadratic Slope	-592.02	175.47	-3.37	<.001	-368.30	171.36	-2.15	<.05
IA-only × Cubic Slope	976.12	344.81	2.83	<.01	592.34	335.65	1.77	.08
Comorbid	0.89	0.34	2.60	<.01	0.74	0.47	1.56	.12
Comorbid × Linear Slope	-21.01	12.48	-1.68	.09	-26.81	12.75	-2.10	<.05
Comorbid × Quadratic Slope	231.85	101.80	2.28	<.05	268.60	99.64	2.70	<.01
Comorbid × Cubic Slope	-503.95	200.51	-2.51	<.05	-531.68	195.93	-2.71	<.01

We repeated the procedure in analyses of treatment trajectories of levels of stress, suicide intent, and urge to use drugs or alcohol. Adding group as a main effect increased model fit for level of stress (*X^2 = ^* 49.99, *df*=8, ΔAIC=34, Δ-2LL=25, *p*<.001) and urge to use drugs and alcohol (*X^2 = ^* 17.16, *df*=8, ΔAIC=1, Δ-2LL=8.6, *p*<.05) but not for suicide intent. For the trajectory of level of stress, the IA only group significantly differed from the ED only subgroup overall (B=-.92, *p*<.001), and the Comorbid group interacted with quadratic and cubic slopes (B=38.53 *p*<.05; B=-60.92, *p*<.01 respectively). For the trajectory of urge to use drugs or alcohol, the IA only group significantly differed from the ED only subgroup overall as well (B=-.68, *p*<.001), not neither groups interacted with slopes significantly.

**Figure 1:**
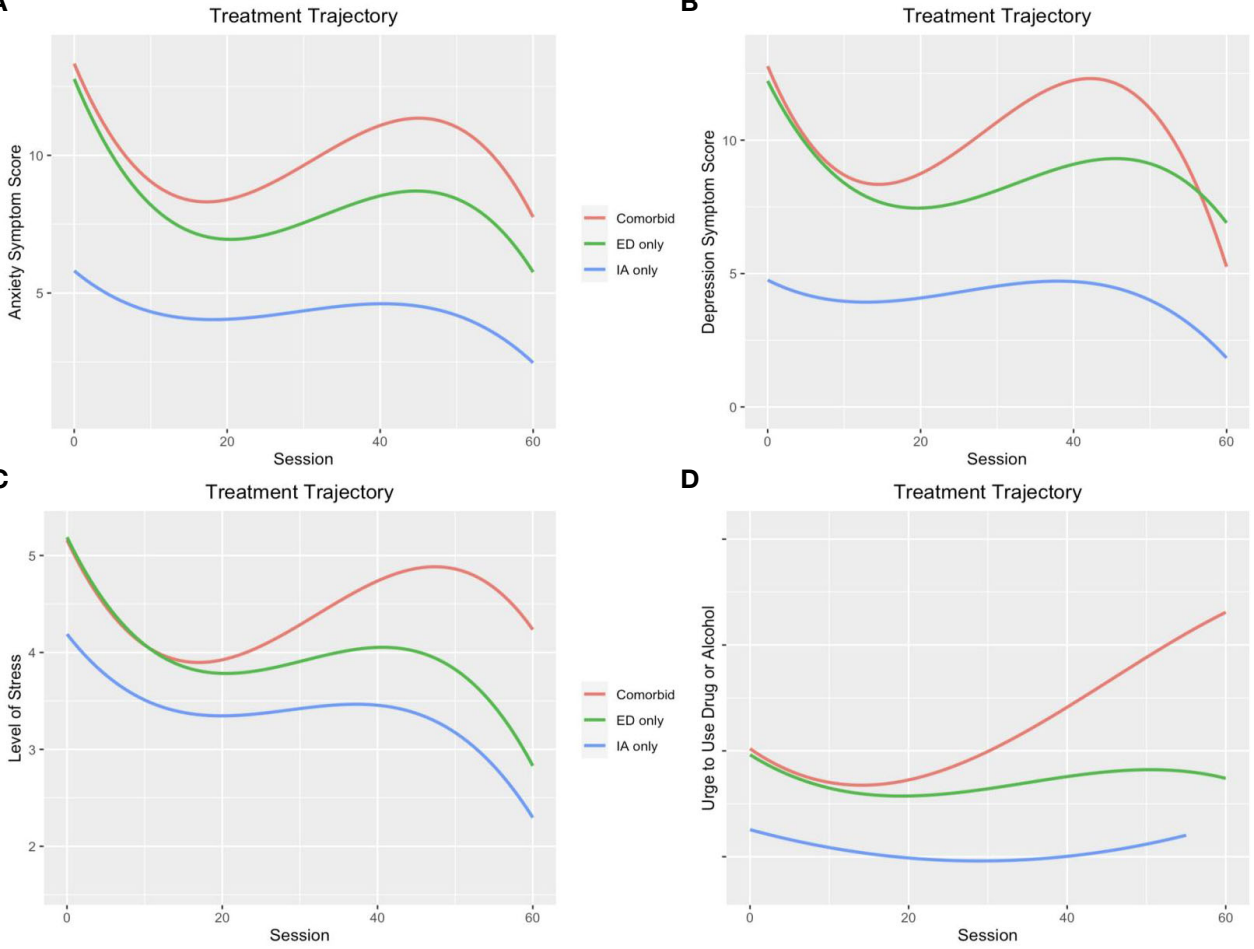
Treatment trajectories of **(A)** anxiety symptoms, **(B)** depression symptoms, **(C)** level of stress, and **(D)** urge to use drug and alcohol across groups of patients with only internet addiction, only emotional disorders, and comorbidity of the two disorders.

**Figure 1** show the symptom change trajectory of the three subgroups over the treatment course. Patients in the IA only subgroup not only reported the lowest levels of anxiety, depression, stress, and urge to use drugs and alcohol at intake, their symptom levels stayed significantly lower than those in the other two groups throughout the treatment trajectory. The ED only subgroup followed the typical cubic trajectory of an initial period of rapid decline of symptoms followed by a longer period of slower improvement. For the Comorbid subgroup, patients display similar trajectory to that of the ED only subgroup but showed a temporary steeper symptom relapse trend before a final decline in symptoms of anxiety, depression, and stress compared to the ED only group. Comorbid subgroup also showed a steady increase in urge to use drugs and alcohol following its initial short decrease.

It is essential to recognize that the collection of our data spanned from 2018 to 2022, an interval that includes the extraordinary circumstances of the COVID-19 pandemic. The pandemic is widely understood to have altered internet usage behaviors and to have had a significant impact on mental health (43). To assess whether the unique pandemic conditions may have influenced our study results, we introduced a binary variable based on a March 2020 cutoff. Statistical analysis revealed that including this temporal variable did not significantly improve the fit of our predictive models, neither was it a significant predictor of the trajectories of treatment outcomes.

## Discussion

This study aimed to explore the differences in pre-treatment factors and treatment process of three groups of patients with IA only, ED only, and comorbidity between the two. Our findings supported the hypothesis that patients with IA only displayed the highest level of functionality compared to the other groups. Specifically, in line with prior research showing the lack of consistent association between IA and life satisfaction (19, 20), we found that IA only group showed a higher level of life satisfaction relative to the other two groups. In addition, the IA only group also reported a more frequent use of cognitive reappraisal as an adaptive strategy to regulate emotion than the other groups, which may further explain their higher level of life satisfaction at intake. Moreover, IA only group reported a much lower level of anxiety and depressive symptoms than the other two groups throughout the treatment trajectory. This finding is consistent with prior studies reporting efficacy and effectiveness of CBT treatment for IA (28).

The study yielded contradictory findings concerning the functionality of the IA only group. When considering the whole sample, IA only group also displayed the highest level of urge to use drug or alcohol, as well as a higher level of anger, urge to harm self, and suicide intent compared to the ED only group. These elevated levels of urges for risky behaviors might be a result of impulsivity traits for patients with IA, as impulsivity is not only a shared criterion across different proposals for IA diagnosis (11–14), but also highly associated with risky urges listed above (for reviews, see 44, 45). The urges themselves as well as their consequences may be the main motives for patients to initiate therapy despite their low level of ED symptoms as well as relative high level of life satisfaction at intake. Conversely, upon analysis of a more engaged subset of the sample—those more consistent in attending sessions and completing survey assessments—the IA only group demonstrated better functionality with respect to stress, anger, suicidal ideation, and the urge to use drugs or alcohol. This discrepancy implies that individuals with IA who adhere more strictly to therapy protocols may have greater functional stability than those presenting with only ED or with comorbid conditions.

Our findings also supported our second hypothesis that patients with comorbidity reported the lowest level of functionality. Not only did they show lower levels of ED symptoms and life satisfaction than IA only group, higher levels of anger, urges to harm self and others, and suicide intent than ED only group, but also had the least use of adaptive emotion regulation among all groups. In addition, patients in the Comorbid group showed a more blunted reduction in depressive and anxiety symptoms and stress compared with those in the ED only group. These results are consistent with prior research showing that depressive cases with higher level of IA were less responsive to treatments compared with cases with lower level of IA (31). Our findings may be attributed to the presence of malfunctioning factors associated with both IA and ED in individuals with comorbidity, resulting in a cumulative effect that surpasses the impact of each disorder in isolation.

It is crucial to highlight the significant rise in anxiety and depressive symptoms and level of stress, as well as a steady increase in urge to use drugs and alcohol observed within the Comorbid group midway through the treatment. One possibility to explain this finding is that as these patients experienced initial lift of IA symptoms, they became more aware of underlying emotional and psychological issues that were previously masked by their excessive internet use. This newfound awareness and emotional sensitivity may contribute to an increase in anxiety and depressive symptoms. If treatment continued to focus on IA-related issues at this stage, the emerging ED symptoms may exacerbate due to a misalignment between the patients’ evolving needs and the therapeutic approach being used. For example, research into methadone maintenance therapy for heroin dependency revealed a sharp improvement in depressive symptoms within the first three months, followed by a more gradual reduction thereafter (46). Prior literature on treatment of substance abuse with comorbid disorders also consistently point out that treatment is most effective when both the substance abuse behaviors and the underlying depression/anxiety are addressed (for a review, see 47). Our findings resonate with prior research, underscoring a need for a comprehensive treatment approach that addresses both IA symptoms and the underlying psychological issues in order to effectively reduce anxiety and depressive symptoms in the comorbid group.

The differences in pre-treatment factors and treatment process of three groups of patients also provide important insight about the discreteness of IA as a potential psychological disorder. First of all, the disparity in patient numbers in the IA only group implies IA is rarer than ED, though frequent internet use is common. Secondly, the findings that IA differed from ED on multiple pre-treatment factors as well as treatment trajectory indicate that IA may be a discrete psychological disorder from depression and anxiety. Thirdly, the IA only and Comorbid groups also exhibited distinct pre-treatment factors and treatment trajectories, pointing to a heterogeneous etiology of IA development. Billieux and colleagues (48) proposed a three pathway theoretical model of the development of problematic phone use that may be applied to general IA. Through the impulsive pathway, individuals with high impulsivity and low self-control can also develop IA due to difficulty disengaging from internet use, and through the extroversion pathway, individuals with high needs of sensation and reward seeking may overuse the internet to access various sources of social and sensory stimulation. In contrast to these pathways based on impulsivity and extroversion, the excessive reassurance pathway applies to individuals with traits of emotional instability, who may rely on the internet as a way to seek reassurance. Given the role of emotional instability, developing IA through the excessive reassurance pathway may render comorbidity with ED more likely than the other two pathways. Future research can further examine the potential heterogeneous pathways as well as prognosis of IA development.

It is pertinent to acknowledge that our findings may not have sufficiently highlighted the full breadth of research exploring the potential interconnections between IA and ED, as other psychological constructs typically associated with ED such as loneliness and insecure attachment have also been found related to IA (49, 50). These transdiagnostic factors play a significant role in understanding the nuanced relationships between IA and emotional dysregulation, potentially serving as underlying mechanisms for the development and maintenance of addictive online behavior as well as emotion disorder. For instance, loneliness could both lead to and be followed by increased online activities (e.g., 51, 52), while insecure attachment may prompt individuals to use the Internet as a coping mechanism to alleviate feelings of social isolation or anxiety (53). Future studies would benefit from a broader inclusion and consideration of these psychological constructs to enrich the understanding of IA as a potentially independent disorder or a secondary manifestation of other existing conditions.

Finally, it is critical to emphasize that all the findings regarding the treatment trajectories were not significantly altered by the pandemic, even though it has been associated with increased internet usage and decreased general well-being (43, 54). The results thus suggest that the behaviors and outcomes we attributed to IA are indicative of its intrinsic properties as a condition, rather than being predominantly driven by the extraordinary circumstances of the COVID-19 pandemic.

### Implications and limitations

Our findings have important clinical implications. First, our findings highlights the need for specialized assessment and treatment approaches for IA. Clinicians should be aware that individuals presenting with IA may have unique characteristics and treatment needs compared to those with ED. Specifically, the elevated levels of risky urges, such as the urge to use drugs and alcohol, among the IA only group indicate a need for targeted interventions addressing impulsivity and risk-taking behaviors. Substance abuse prevention and harm reduction strategies should be integrated into treatment plans for individuals with IA who exhibit these risky urges. Second, the slower reduction in depressive and anxiety symptoms observed in the Comorbid group compared to the ED only group suggests that comorbidity between IA and ED may be associated with poorer treatment outcomes. Clinicians should be prepared to address both IA and ED symptoms concurrently, as targeting one disorder alone may not be sufficient for optimal treatment response.

The results of the study should be considered in the context of the following limitations. First, to facilitate ease of completion, the session questionnaire was intentionally designed to be succinct; as a result, our study did not incorporate a longitudinal assessment of IA symptomatology during the course of treatment. This omission limits our ability to trace the trajectory of IA symptoms over time and evaluate their possible attenuation or exacerbation in response to therapeutic interventions. Relatedly, while YDQ is widely recognized and utilized in research due to its brevity and ease of administration, it may not encompass the full spectrum of IA’s complexities, potentially leading to significant interpretative errors and biases by measuring aspects that may not constitute the core of IA. A deeper and more accurate understanding of how individuals’ IA symptoms change in the course of treatment could provide valuable insights into the interplay between IA and co-occurring conditions such as depression. Second, the study fell short in assessing the specific content of Internet activities that participants engaged in – a factor that could significantly influence the severity and impact of IA. Without categorizing IA behaviors in a granulated manner, we were unable to conclusively determine how different online behaviors contribute to the pathology of IA and intersect with depressive symptoms. This broader understanding could potentially catalyze the development of targeted therapeutic strategies. Third, the number of therapy sessions reported in our study does not accurately encompass the entirety of the therapeutic engagements. Due to instances where session surveys were not completed, there is a discrepancy between the recorded data and the actual number of sessions that took place. This reporting gap may obscure the dose-response relationship between therapy and clinical outcomes and could potentially lead to a misinterpretation of treatment effects. Furthermore, our research did not capture data regarding the extent to which individual therapy sessions directly addressed IA. Understanding whether and how IA was targeted in therapy is crucial for assessing treatment effectiveness. In the absence of such information, it is challenging to gauge the impact of psychotherapeutic interventions on IA and compare it to the impact on ED symptoms. As such, we may miss out on identifying potentially pivotal therapeutic moments or interventions that could be particularly beneficial for patients with comorbid IA and depression. Lastly, while the manuscript identifies a few critical variables in the investigation of IA as a discrete condition, it falls short in the detailed exploration of potential common etiological factors, such as insecure attachment styles or loneliness, which merit further examination in the context of IA. By recognizing these limitations, we aim to set a clear agenda for future research to extend and refine the understanding of IA, its diagnosis, and its treatment.

In conclusion, our study adds to the growing body of literature on IA and its relationship with ED. The findings support the notion of IA as a distinct psychological disorder and highlight the need for tailored assessment and treatment approaches for individuals with IA with and without ED comorbidity.

## Data availability statement

The datasets presented in this article contain clinical information and are not readily available to protect confidentiality. Requests to access the data can be directed to the corresponding author.

## Ethics statement

The studies involving humans were approved by the Institutional Review Board of Touro College (IRB Exempt Protocol Number: IRB1-2023-003). The studies were conducted in accordance with the local legislation and institutional requirements. The ethics committee/institutional review board waived the requirement of written informed consent for participation from the participants or the participants’ legal guardians/next of kin because research using unidentifiable data.

## Author contributions

JZ: Writing – review & editing, Writing – original draft, Methodology. DR: Writing – review & editing. SP: Writing – review & editing, Methodology.
